# Degradation and Recycling of Films Based on Biodegradable Polymers: A Short Review

**DOI:** 10.3390/polym11040651

**Published:** 2019-04-09

**Authors:** Roberto Scaffaro, Andrea Maio, Fiorenza Sutera, Emmanuel Fortunato Gulino, Marco Morreale

**Affiliations:** 1 Department of Engineering, Viale delle Scienze, University of Palermo, 90128 Palermo, Italy; fiorenza.sutera@unipa.it (F.S.); emmanuelfortunato.gulino@unipa.it (E.F.G.); 2 Faculty of Engineering and Architecture, Kore University of Enna, Cittadella Universitaria, 94100 Enna, Italy; marco.morreale@unikore.it

**Keywords:** biodegradable polymers, films, degradation, recycling, multi-layer, coextrusion

## Abstract

The environmental performance of biodegradable materials has attracted attention from the academic and the industrial research over the recent years. Currently, degradation behavior and possible recyclability features, as well as actual recycling paths of such systems, are crucial to give them both durability and eco-sustainability. This paper presents a review of the degradation behaviour of biodegradable polymers and related composites, with particular concern for multi-layer films. The processing of biodegradable polymeric films and the manufacturing and properties of multilayer films based on biodegradable polymers will be discussed. The results and data collected show that: poly-lactic acid (PLA), poly-butylene adipate-co-terephthalate (PBAT) and poly-caprolactone (PCL) are the most used biodegradable polymers, but are prone to hydrolytic degradation during processing; environmental degradation is favored by enzymes, and can take place within weeks, while in water it can take from months to years; thermal degradation during recycling basically follows a hydrolytic path, due to moisture and high temperatures (β-scissions and transesterification) which may compromise processing and recycling; ultraviolet (UV) and thermal stabilization can be adequately performed using suitable stabilizers.

## 1. Introduction

Polymeric films attract a significant degree of interest because of their widespread use in several industrial applications, with particular reference to packaging. Over the last decades, the large use of oil-derived polymers, due to their good mechanical, thermal and barrier properties, as well as their low cost, has led to significant problems in term of production and accumulation of wastes [1,2]. Rising concern about the reduction of waste coming from plastic packaging has encouraged both academia and industry to engage in research focused on polymers coming from natural resources, biodegradable or compostable [3,4,5,6]. Bio-based polymers are those obtained from natural resources, while biodegradable polymers are those which can degrade into water and carbon dioxide under normal environmental conditions through microbial action, providing compost as a simple and sustainable disposal option [7,8,9,10], and all of these can be regarded to as bioplastics. For instance, in the case of food packaging, polymers combining renewable sources and biodegradable nature are preferred [11,12].

The replacement of traditional packaging requires the new biodegradable packaging to guarantee comparable levels of performance and cost [13]. However, bio-based films often show disappointing mechanical, thermal and barrier properties [14]. Therefore, new strategies for the development of materials with suitable properties to replace traditional materials for packaging have attracted many research efforts [15,16,17]. 

Several biodegradable polymers were developed and many of them showed good potential for several applications. Among them, poly-lactic acid (PLA) probably attracted the greatest interest for several applications; in fact, being bioresorbable and biocompatible, this polyester is mainly employed in biomedicine for the preparation of tissue-engineering scaffolds, drug-delivery devices, biosensors, and is considered one of the most promising for food-packaging applications [18]. It is versatile, compostable, recyclable [19,20,21,22], highly transparent, with a high molecular weight, high resistance to water and good processability; on the other hand, its mechanical and thermal properties are still not suitable for several applications [23,24]. 

Among the polyesters, poly-caprolactone (PCL) has traditionally found applications in the fields of biomedicine and food packaging. With respect to PLA, it presents lower stiffness and tensile strength but higher stretchability and better processability. Moreover, the temperature required for its melt compounding (below 100 °C) allows it to be incorporated in a broader spectrum of natural additives for food packaging, especially those prone to thermal degradation/deactivation [25].

Poly-butylene adipate-co-terephthalate (PBAT) belongs to biodegradable aliphatic-aromatic polyesters and, thanks to its high ductility, it finds applications in agricultural or food packaging materials and was recently considered as one of the candidate materials for blending with PLA [26,27].

Another promising biodegradable polymer is the MaterBi^®^ or, better, the MaterBi^®^ family, including a series of bioplastics coming from modified starch and/or synthetic biodegradable polyesters [28,29]. It found applications in several fields due to good mechanical properties, thermal stability, barrier properties, as well as good processability and compostability [30,31,32]. Another recent example of biodegradable/compostable polymer is the Bioflex^®^ family, typically based on biodegradable polymers such as PLA and thermoplastic copolyesters, such as PBAT [33,34,35,36,37].

Other examples of sustainable green polyesters are poly(glycolic acid) (PGA), poly(butylene succinate) (PBS), and poly-hydroxyalkanoates (PHA). PGA finds application in biomedicine owing to its ability to increase the biocompatibility and ductility of PLA, whereas PBS is typically used for film packaging [38,39]; however, it has been proposed even for tissue engineering in combination with chitosan [40].

Chitosan, a polysaccharide constituted by randomly distributed β-(1-4)-linked D-glucosamine units, attracts significant interest in a broad variety of areas, ranging from medicine and related fields, to environmental protection, including also food science or agriculture, by virtue of its biodegradability, biocompatibility, antimicrobial activity, adsorption capability and chelating properties [41]. However, this biopolymer displays unsatisfactory mechanical properties, hence it is often used in combination with other polymers and additives [41]. 

Among the biopolymers, natural polysaccharides, such as cellulose and starch, and proteins, including zein and silk fibroin, find applications in food packaging, being ecofriendly and fully biodegradable. Furthermore, some of them may be particularly suitable for triggering the release of natural antioxidants, such as polyphenols, useful for increasing the shelf-life of foodstuffs [25].

In this brief review, some general information will be provided with regard to the environmental performance of biodegradable polymers and biodegradable polymer-made systems, with concern to their degradation behavior and their possible recyclability features, and actual recycling paths; subsequently, an overview specifically focused on the processing of biodegradable polymer-based films and on the manufacturing and properties of biodegradable polymer-based multilayer films will be discussed. There will be no focus on biomedical applications, since it would be out of the scope of the brief review.

## 2. Degradation of Biodegradable Polymer-Based Systems

Some studies are available regarding the numerous degradation paths of bioplastic-based systems.

**Figure 1:**
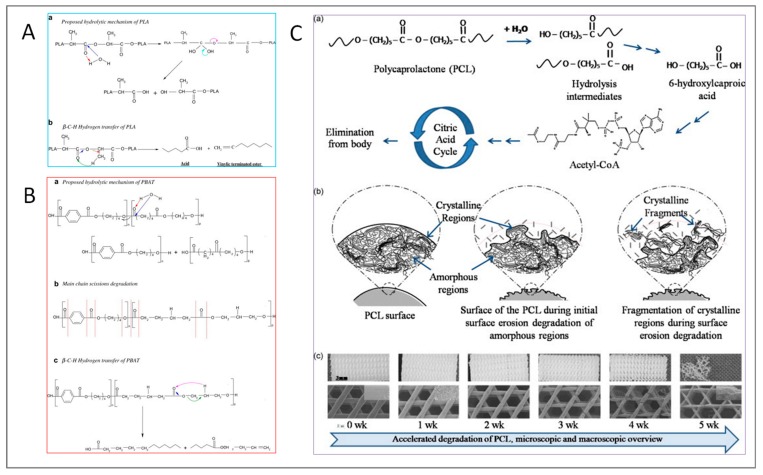
Schematics of hydrolytic degradation mechanisms of the most widespread biodegradable polyesters: poly-lactic acid (PLA) (**A**), poly-butylene adipate-co-terephthalate (PBAT) (**B**) [42], and poly-caprolactone (PCL) (**C**) [43] (reproduced with permission from Elsevier).

Biodegradable polyesters are prone to several degradation mechanisms that may occur depending on the processing conditions. Hydrolysis of ester linkages is a water-induced degradation mechanism, whose rate and extent depends on water concentration, pH, eventual presence of acid or base catalyst, morphology of the polymer and temperature [42]. Figure 1A provides two common degradation pathways proposed for PLA: hydrolytic chain scission (subpanel a) and main chain scission, i.e., β-C-H hydrogen transfer (b). Hydrolytic degradation in PLA can take place during melt processing or in aqueous media. In the former case, it can be activated by the presence of moisture at high temperatures, in the latter, hydrolytic reactions display pH-dependent kinetics, being particularly faster under alkaline conditions. In both cases, the degradation determines a dramatic molecular weight reduction and can be easily monitored by spectroscopic measurement of –OH and –COOH groups, since the degradation products are hydroxyl- or carboxyl- terminated PLA. In the case of β-C-H hydrogen transfer, instead, carboxylic acid end groups and vinyl esters are formed. Moreover, at high temperatures (above 200 °C), the main degradation mechanism of PLA is trans-esterification, which leads to the formation of cyclic oligomers. 

Similarly, PBAT undergoes hydrolytic degradation owing to the cleavage of ester linkages (Figure 1B, subpanel a), which requires the presence of water. However, in the case of PBAT, H_2_O can even react with the carbonyl groups located in proximity of benzene rings (subpanel b). β-C-H hydrogen transfer reactions are supposed to occur randomly even in PBAT backbone [42].

PCL, whose typical degradation pathway is depicted in Figure 1C, substantially follows the same mechanisms as those previously discussed for PLA and PBAT, although its higher crystallinity degree results in slower degradation [43].

Hydrolytic degradation of poly(α-hydroxyl) esters can proceed by either surface or bulk degradation pathways, with these latter being regulated by mass transfer phenomena (diffusion) and kinetics of chemical reactions [43]. If the rate of hydrolytic chain scission is faster than that of water diffusion into the polymer bulk, the hydrolysis occurs only at the polymer–liquid interface, thus resulting in the progressive thinning of the sample, whose bulk properties, in terms of molecular weight and crystallinity, remain unaltered. Otherwise, when water diffusion is faster than hydrolytic reactions, hydrolysis takes place randomly throughout the entire polymer bulk. This aspect leads to an overall and uniform decay of molecular weight. Oligomers and monomers that are formed diffuse out, thus causing gradual erosive phenomena until achieving the equilibrium between diffusion and chemical kinetics. Whether this equilibrium is hindered, the accumulation of -OH and -COOH terminated byproducts may trigger an internal autocatalysis that accelerates the bulk degradation with respect to that of the outer layers. In this case, a bimodal distribution of molecular weights would be observed, with a degraded inner core and a less altered skin. As the oligomers become small enough to diffuse throughout the structure, this latter tends to be hollowed. 

Enzymatic biodegradation of PCL and PLA is shown to be extremely fast when accomplished by outdoor living organisms (bacteria and fungi), typically present in the soil, whereas biodegradation in the human body, for instance, due to the lack of suitable enzymes, is extremely slow, ranging from 6–12 months for PLA to 2–4 years for PCL, depending on starting crystallinity and molecular weight [44]. In fact, bio-resorbability of these polymers involves a two-stage mechanism: hydrolysis of ester groups occurs in the first stage, while the intracellular digestion is carried out by macrophages only in a subsequent stage, that is when the polymer molecular weight is low enough, and crystallinity degree is extremely high [45].

Singh et al. [46] prepared a review, discussing different kinds of polymeric degradations along with their mechanisms, including thermal degradation, photo-oxidative degradation, catalytic degradation, biodegradation, mechanic-chemical degradation and ozone-induced degradation, highlighting how different polymers can behave in a consequently different way according to the actual degradation environment and the mechanisms involved. This was confirmed also by the studies of Ramasubramanian [47] in his review on polymer degradation mechanisms, providing also some possible parameters affecting the degradation process and stating that the degradation process is affected not only by molecular weight, chemical structure and bond type, but also by the nature and mechanism of the degradation process.

In the following, more studies regarding degradation of specific systems are reported, on the basis of the biodegradable polymers used.

Sarai Agustin-Salazar et al. [48] investigated on the potential of pecan (*Carya illinoinensis*) nutshell (NS) as a source of antioxidants, with particular reference to the thermo- and photo-oxidative stability of PLA and polyethylene (PE). Several phenolic constituents, including proanthocyanidins, were found in NSE and their actual radical scavenging action was assessed. They found that NSE acted as a thermal stabilizer for PLA and PE films. Under ultraviolet (UV) irradiation, NSE was found to be more effective in protecting PE rather than PLA, mainly because of a combination of peroxy radical scavenging and inhibition of Norrish-type photolytic cleavage. 

Fukushima et al. [49] studied the biodegradation of neat poly(DL-lactide) (PDLLA), PCL and a partially miscible PDLLA/PCL blend in compost, up to 12 weeks. They found that the PCL proved to be stable against abiotic hydrolysis due to its semicrystalline structure and hydrophobicity, in agreement with the previously reported discussion on the degradation mechanisms of PCL, while the PDLLA degraded quite fast due to its amorphous nature. With regard to the blend, an interesting result was found: its continuous PDLLA phase accelerated the hydrolysis of the PCL phase. In compost, the degradation kinetics appeared to be generally faster. 

Mofokeng et al. [50] studied the thermal stability of PLA/PCL blend nanocomposites containing TiO_2_, by means of thermogravimetric analysis (TGA) and Fourier-transform infrared (FT–IR) spectroscopy. The neat PCL showed better thermal stability but, on the other hand, neat PLA showed to a higher activation energy of degradation: this suggests that the degradation rate is more temperature-dependent, probably due to a degradation mechanism based on chain scission and re-formation. However, the PLA/PCL blend showed a reduced thermal stability, although the addition of TiO_2_ nanoparticles helped to improve it. 

Castilla-Cortazar et al. [51] investigated on long-term hydrolytic and enzymatic degradation paths of PCL networks. The hydrolytic degradation studies were carried out both in water and in phosphate buffer solution (PBS), finding higher degradation rates than in the case of linear PCL (weight loss approximately 20% in 60 weeks) after immersing the samples either in water or in PBS conditions; similar findings were reported for enzymatic (Pseudomonas Lipase) degradation, although the 20% weight loss was reached in a shorter time (14 weeks). However, the changes in some properties, such as crystallinity, crystal size and elastic modulus were smaller in the latter case, thus suggesting significant differences between the two degradation mechanisms (hydrolytic and enzymatic), in agreement with the previously reported studies.

Tsutsumi et al. [52] studied the actual degradability, both enzymatic (induced by lipases) and chemical (in NaOH solution) of several biodegradable polyesters, such as poly(butylene succinate adipate) (PBSA), poly(butylene succinate) (PBS), poly(ethylene succinate) (PES), poly(butylene succinate)/poly(caprolactone) blend and PBAT. In enzymatic degradation, PBSA was shown to be significantly degradable, while PBAT was not degraded much by several kinds of lipases, due to its aromatic ring. In NaOH solution, the degradation rate of PES was quite faster than in the other polyesters; furthermore, degradation rate of PBSA in such conditions was lower than in enzymatic degradation. 

Nikolic et al. [53] provided information on biodegradable PBAT in quite different environments, finding a faster degradation rate when buried in soil rather than under laboratory conditions (in a Xenon tester); for instance, the elongation at break dropped from 900% to 70% in one month, versus 6 months in laboratory. Most of these changes were attributed to surface morphology modifications (cracks and holes), suggesting that the enzyme-related hydrolysis of PBAT is actually controlled by surface mechanisms, especially in the presence of fungi rather than bacteria. As regards the UV degradation, it was found to be controlled by the photochemistry of the terephthalate moiety.

Touchaleaume et al. [54] provided further insight regarding the degradation paths of PBAT-blends, testing biodegradable films (respectively, PBAT/PLA, PBAT/PPC and PBAT/Starch) in real field conditions, i.e., on the soil or buried in the soil, thus investigating on the actual role of UV radiation and microorganisms. Indeed, it was found that biodegradation could occur onto the soil, but the early stages were mainly attributed to UV radiations. Regardless of the different materials tested (although the main component was, in all cases, PBAT, therefore it could be expected that the other components may not have significant impacts on the degradation process), the worsening of mechanical properties was always correlated with crosslinking phenomena of the PBAT fraction and consequent formation of a gel fraction. 

The latter studies, therefore, confirm the importance of surface mechanisms in the control of degradation kinetics of PBAT. 

Sangroniz et al. [55] analyzed the effect of adding a non-degradable component, poly(hydroxy ether of bisphenol A) (PH), to the degradation of biodegradable PBAT. Blends were prepared by different methods and, depending on this, miscible or partially miscible blends were obtained. In the case of miscible blends, the authors carried out the hydrolytic degradation for approximately 400 days, finding that the adipate sequences on PBAT copolymer are more prone to degradation. The degradation rate was found to decrease slightly in the presence of PH, depending on its amount, thus suggesting a way to tailor the actual degradability. 

As regards the behaviour of other biodegradable polymers such as MaterBi^®^ in a marine environment (i.e., a condition which is currently of topical interest by the mass-media), the study from Muller et al. [56] should be cited. They took into account the decay behaviour of three types of shopping bag polymers (standard, degradable, and biodegradable plastic) in sea turtle gastrointestinal fluids. In particular, a standard bag was based on HDPE, degradable bag was made of a proprietary material composed of either PE or PP with additives, while a biodegradable bag was based on MaterBi^®^. Over a 49-day observation period, only negligible degradation rates (based on mass loss) were found for the standard and the degradable plastic, as expected; on the other hand, up to 9% was found for the biodegradable bag, lower than the degradation rate typically expected for industrial composting conditions.

## 3. Recycling of Systems Based on Biodegradable Polymers

As previously described, the use of bioplastic-based packaging allows significant advantages to be obtained in terms of environmental impact related to the entire life cycle of the product [57,58,59,60]. However, this can be further improved if the post-consumption recycling of bioplastic-based systems is carried out [61,62,63,64,65,66,67].

The idea to recycle biodegradable polymers, currently used for several applications [13,65,68,69] may sound odd, considering that polymers are regarded as a sustainable alternative for oil-derived polymers since they come from renewable resources and since they are biodegradable/compostable; however, there are several reasons to suggest that recycling of bioplastics is a sensible strategy. These are mainly related to the growing industrial demand and to the fact that recycling is crucial to the reduction of non-renewable resources consumption (including the energy demand linked with their production). Furthermore, some commercial bioplastics do not undergo severe degradation under normal conditions, and the disposal of bioplastic-made items leads to the disposal of valuable raw secondary materials [70,71,72,73]. 

Several studies have investigated the possibilities of recycling biodegradable polymers in order to reduce the environmental impacts related to their life-cycle [61,62,64,66,74,75]; particular attention has been focused on PLA [61,62,64,76]. A previous review [71] focused on mechanical recycling of PLA; it was shown, in agreement with other studies [77,78] that mechanical recycling of PLA is a simple and cost-effective recycling approach. 

Moreover, in view of the rising interest towards polymer nanocomposites, due to their improved mechanical thermal and barrier properties [79,80], some researchers have recently studied the of recycling biobased nanocomposites [19,63,81]. With particular reference to PLA, it is known that this polymer undergoes thermodegradation during melt processing [76,82] and, therefore, its recyclability is strictly related to the actual extent of thermodegradative phenomena during melt processing [76,83,84]. For instance, Tesfaye et al. [81] investigated the effect of silk nanocrystals (SNC) on thermal and rheological properties of PLA subjected to repeated extrusion operations; they found that the presence of SNC slows down the thermal degradation of PLA. Peinado et al. [63] studied the effects of extrusion on the rheological and mechanical properties of PLA filled with nanoclays; they found that, although both PLA and nanocomposites show viscosity decreases after each processing step, there was no major reduction of the mechanical performance.

It was generally observed that upon reprocessing cycles, the properties of biodegradable-based nanocomposites depends on to two opposite effects: (i) chain scission due to thermo-mechanical degradation, and (ii) filler dispersion effect resulting from multiple processing. Although the latter phenomenon may prevail at low reprocessing cycles, the former proves to be dominant at higher cycles. Among possible strategies were proposed the reactive extrusion with chain extenders or branching agents [42] or using stabilizers [85]. However, even a combination of chain extenders and stabilizers may be involved to increase the recyclability and reprocessability of biodegradable polymers.

## 4. Processing of Films Based on Biodegradable Polymers

As briefly discussed in the introduction, it is of significant interest to investigate the processability of biodegradable polymers, especially with concern to the manufacturing of films. The scientific literature reports several studies, which can provide an overview of this specific issue.

Puccini et al. [86] prepared flexible films from polyethylene and hydrolyzed collagen (HC) via film blowing. HC content was between 10% and 50%, and average thickness was 60 μm. Furthermore, maleic anhydride functionalized ethylene elastomers were used as compatibilizers. It was found that compatibilized showed interesting mechanical and thermal properties, suggesting a possible suitability to applications in packaging and agriculture. 

Li et al. [87,88] prepared films from poly(lactic acid)/poly(butylene adipate-co-terephthalate) (PLA/PBAT) blends via melt blending and film blowing, also using a commercial chain extender containing epoxy functional groups and investigating the effect of the latter on the mechanical, rheological, morphological and thermal properties. They found significant improvement of mechanical properties such as elongation at break, tensile strength and tear strength upon using a small amount of the chain extender; scanning electron microscopy (SEM) analysis confirmed an enhanced compatibility due to its presence. Furthermore, the sealing strength of PLA/PBAT/ADR film was evaluated and found to be higher than that of PLA/PBAT, suggesting suitability for shopping bags applications. 

Mallegni et al. [89] prepared blown films from PLA/PBAT and PLA/PBS blends in the presence of polypropylene glycol di glycidyl ether as plasticizer and compatibilizer. The processability was adequate and the tearing strength showed a maximum like trend in the investigated composition range, with the tensile strength in the range 12–24 MPa, the elongation at break in the range 150–260%.

Li et al. [90] focused on the unsatisfactory water vapor barrier properties of PBAT films, preparing nanocomposites films containing organically modified montmorillonite (OMMT), via film blowing and biaxial orientation. They also compared the results with those predicted on the basis of the Bharadwaj model. It was found that the barrier properties greatly improved on increasing the OMMT percentage, although a plateau was reached; on the other hand, the elastic modulus systematically increased up to the maximum investigated OMMT amount (13 wt %) while the elongation at break reached a maximum (5 wt %) and then dropped. The relationship between clay amount and water vapour permeability was found to be in good agreement with the model, and dependent on the orientation (parallel to film surface) of the clay platelets. 

Gupta et al. [23] focused on the processability and properties of PLA films, using spray-dried, lignin-coated cellulose nanocrystals (CNCs) as fillers and choosing film blowing as a processing technique. They found that lignin coating enhanced the dispersion of CNCs as well as interface adhesion with PLA, leading to significant improvements of the storage modulus even with very low amounts of CNCs (0.3–0.5 wt %), attributed to the very good dispersion which promoted an increased PLA crystallization. 

Ruellan et al. [91] attempted to overcome PLA fragility by using palm oil deodorizer distillate (PODC) as additive. They prepared blends where rigidity and glass transition temperatures were not changed much, while the elongation at break increased significantly; the additive also acted as a processing aid during the film-blowing operation. 

Schneider et al. [92] prepared blown films from PLA and PBAT were produced using an epoxy functionalized-poly(lactide) (EF-PLA) in order to provide some degree of compatibilization (through the epoxy groups reacting with the PBAT) as well as an improvement of the rheological properties. They found that the compatibilizer enhanced the melt strength, thus allowing use of higher PLA amounts (up to 70%) and especially to have higher bubble stability. Furthermore, the typical static charge build-up was reduced. At the same time, mechanical properties such as dart resistance were found to be significantly improved (up to 400%).

Cunha et al. [93] focused on the poor processability of poly(hydroxy butyrate-co-valerate) (PHBV) during film blowing operations, preparing blends with PBAT as secondary component. They found an increased bubble stability and therefore an improved processability. 

Thunwall et al. [94] studied thermoplastic starch blends (based on potato starch, glycerol and water) in order to find the most suitable processing conditions, including temperature, glycerol amount and moisture content in a film blowing operation. Natural starch grade, as well as an oxidized and hydroxypropylated grade, were used. Natural starch processability was significantly more difficult and sticky behavior was one of the major problems encountered. A reasonable compromise was found with approximately 20% glycerol and approximately 10% moisture content. 

La Mantia et al. [37] studied the rheological properties of biodegradable polymers subjected to film-blowing processing, as well as the mechanical properties and the suitability to industrial scale film-blowing operations. The investigated biodegradable polymers were from the MaterBi^®^ and Bioflex^®^ commercial “families”. Also, different blow-up ratios and draw ratios were chosen. The study allowed selecting the most suitable biodegradable polymers from a range of systems coming from the above reported commercial families. 

Sun et al. [95] studied the effect of PHA content on hydroxypropyl distarch phosphate (HPDSP)/PHA films via film blowing. They found that the film crystallinity increased on increasing the PHA content, attaining the highest light transmittance and tensile strength at 12% PHA content, while the water vapor permeability, the elastic modulus, and the thermal resistance decreased upon increasing the PHA wt%. 

Basically, all the studies focused on processing of bioplastics pointed out that during melt compounding, these biodegradable polymers tend to undergo hydrolytic degradation and/or β C-H scission reactions. In this context, some nanocarbon-based fillers could be used to avoid the risk of thermal and chemical oxidation of bioplastics during melt processing [96,97,98,99,100,101] due to their scavenging activity. Another possibility relies on the use of natural antioxidants, such as polyphenols, essential oils, lignin nanocrystals, vitamins, which offer several benefits in terms of safety, health and environmental sustainability [102,103].

## 5. Properties of Biodegradable Polymer-Based Multilayer Films

Among the several strategies available, such as the addition of nanoparticles to the polymer matrices [104,105,106,107,108,109,110,111] or the preparation of polymer blends [112,113], the development of multilayer systems is particularly interesting. 

**Figure 2:**
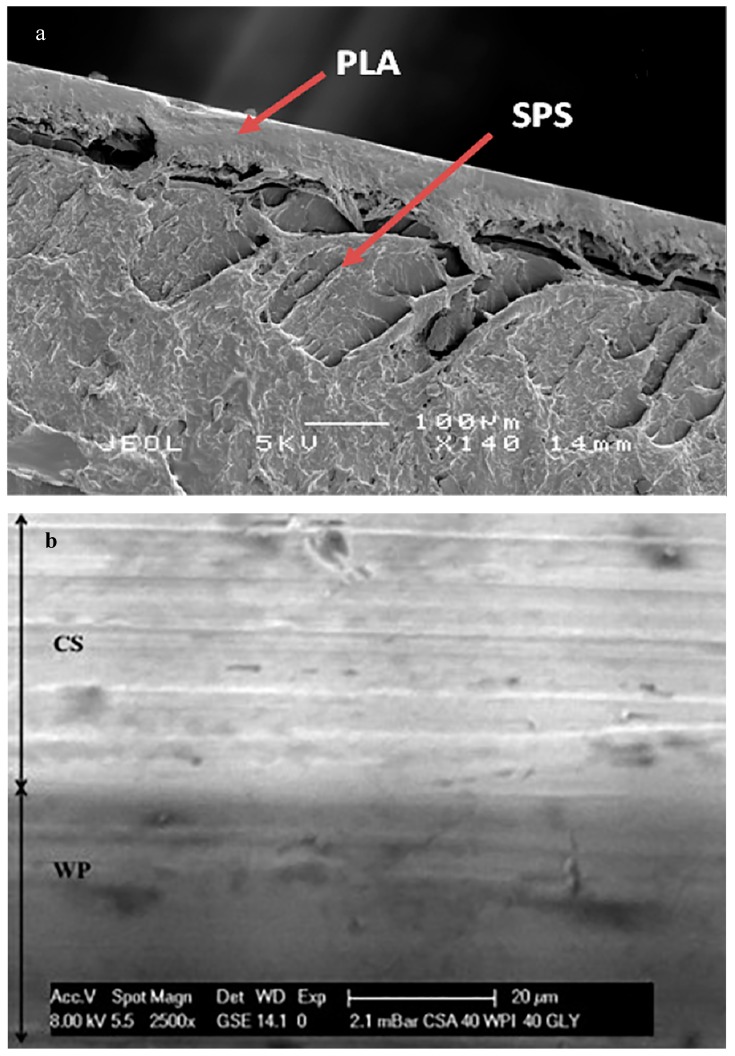
Scanning electron microscope (SEM) images of TD fracture surfaces of (**a**) PLA/sugar-palm starch (SPS) film [18] and (**b**) chitosan/whey protein (CS/WP) film [116] (reproduced with permission from Elsevier).

Recently, Sanyang et al. [114] developed environment-friendly bilayer films from sugar-palm starch and polylactic acid (PLA) using solvent-based methods. Morphological analysis showed a lack of interface adhesion between the two layers (Figure 2a), and mechanical tests also pointed out a relatively poor tensile resistance, although it was higher than that of neat sugar-palm starch (SPS) films.

Gonzalez et al. [115] used a solvent-casting method in order to prepare a PLA-soy protein (SPI) bilayer film. In this case, a good layer-layer adhesion was found, as well as interesting mechanical properties. 

Kurek et al. [116] developed bilayer films via solution coating, starting from chitosan (CS) and whey protein (WP). The obtained films showed good transparency, improved mechanical performance, and better water barrier properties in comparison to the corresponding monolayer films; Figure 2b also shows that the adhesion between the two layers is good. 

**Figure 3:**
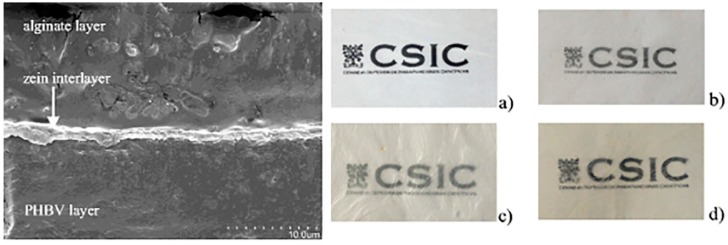
**Left**: SEM micrograph of TD fracture surface, poly(hydroxy butyrate-co-valerate) (PHBV)/zein/alginate film; **right**: images of several multilayer systems: (**a**) PHBV; (**b**) PHBV/zein+CNMA; (**c**) PHBV/zein + CNMA/PHBV; (**d**) PHBV/zein+CNMA/alginate [17] (reproduced with permission from SpringerNature).

Cerqueira et al. [17] prepared active multilayer films by electrospinning a layer of zein (with or without the incorporation of cinnamaldehyde) onto a base film made of PHBV. Then, a third layer (made of PHBV or alginate) was added to the previously described bilayer, by solvent casting. In particular, the assembly of these three-phase systems was performed by hot-pressing. As observable from Figure 3, the SEM micrograph shows a good adhesion between the zein layer and both of the other two (alginate and PHBV). With regard to the optical properties of the film, it was observed that the incorporation of cinnamaldehyde, as well as the presence of the third layer, lowered the overall transparency. The authors also showed that the release of the active component, i.e., cinnamaldehyde, is controlled by the presence and the kind of the third layer. 

**Figure 4:**
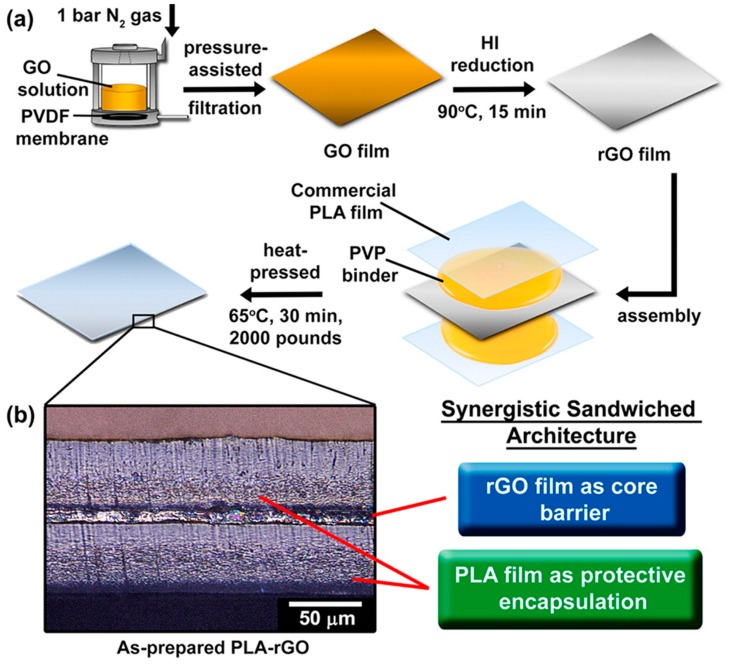
(**a**) Manufacturing scheme of PLA/graphene oxide (GO) film; (**b**) SEM micrograph showing the sandwich-like architecture of PLA/rGO films (reproduced with permission from [16]. Copyright 2016 American Chemical Society).

Goh et al. [16] prepared a biodegradable, multilayer sandwich structure, consisting in two PLA layers with an interposed graphene oxide (GO) layer. The preparation of the sandwich structure was performed by hot pressing, using a polyvinylpyrrolidone (PVP) layer as binder between PLA and GO. Figure 4a shows the preparation scheme while Figure 4b shows the SEM micrograph of the transverse section. The results reported excellent barrier properties to water vapour and oxygen, thanks to the compact lamellar structure of graphene oxide. 

**Figure 5:**
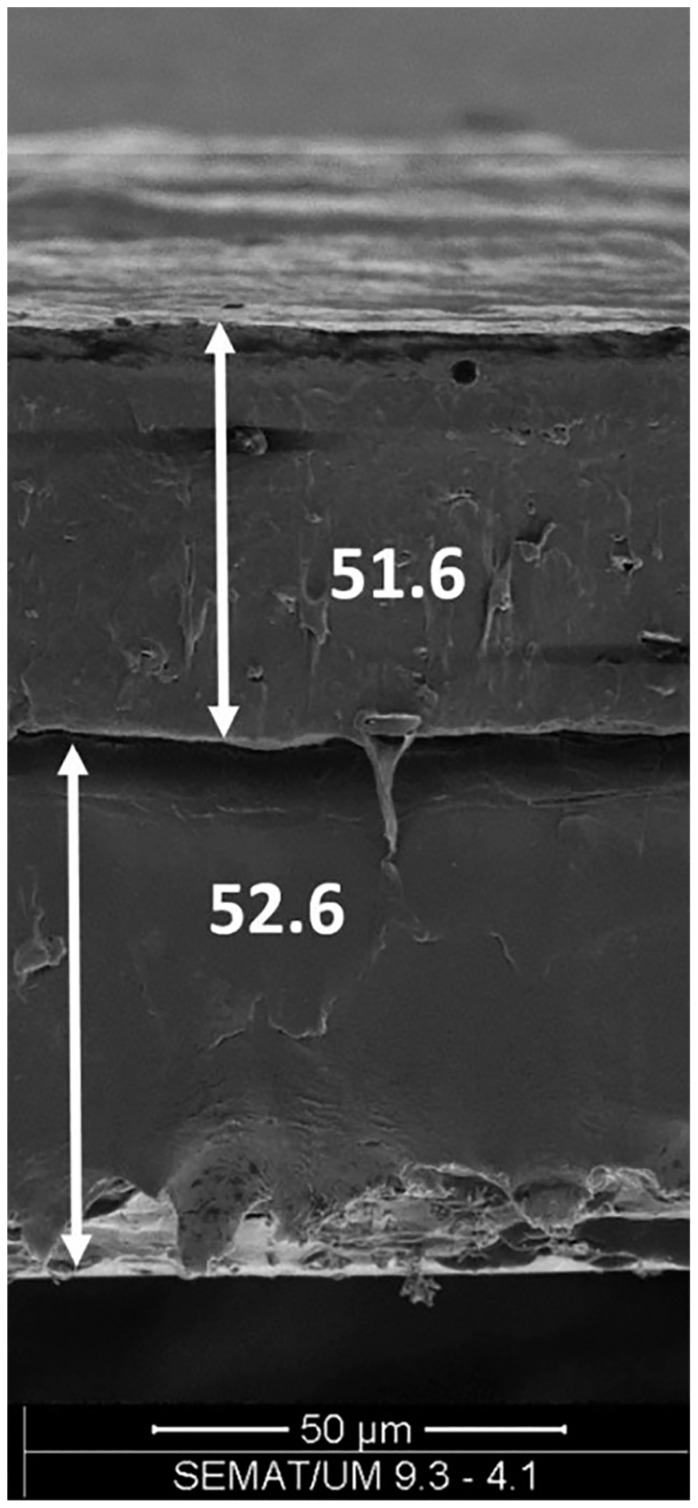
SEM image of bilayer PBAT/PHBV film (thicker layer is PHBV) prepared by Cunha et al. via film blowing [93] (reproduced with permission from John Wiley and Sons).

Unfortunately, the solution-based methods, previously described, are adequate for manufacturing multilayer films only on laboratory scale, but not enough for large-scale production. On the other hand, a suitable technology for mass production is extrusion, and particularly film blowing, which is known to be a highly efficient technique for the industrial production of polymeric films [117]. This continuous process involves different operations at the same time: melting, mixing, stretching, convoying and forming, and is widely used for the industrial manufacturing of monolayer films [118,119,120]. However, it has been widely adopted, so far, for the production of multilayer films of petroleum-derived polymers, while there are only few examples in the scientific literature of film blowing of biodegradable polymers through coextrusion. Cunha et al. [93] prepared a PHBV/PBAT bilayer film and tested its properties, finding similar mechanical properties to commercial PBAT films, although the adhesion between the two layers is unsatisfactory, as noticeable in Figure 5, leading to delamination after a 10% deformation. 

From the above reported works, therefore, one of the main concerns which arise is surely related to the interlayer adhesion that may be promoted by using proper additives or by introducing electrospun layers aiming at limiting the debonding issues. 

Table 1 reports, in a concise and synoptic way, the main data regarding the above reported studies. 

## 6. Conclusions

The main degradation pathways of the most important bioplastics and related systems were discussed. The reports from the literature revealed that PLA, PBAT, and starch can be degraded in real field conditions, i.e., both on the soil and buried in the soil. Biopolymers and bioplastics are prone to degradation by UV radiation and microorganisms. Several biodegradable polyesters proved to undergo both enzymatic (induced by lipases) and chemical (in NaOH solution) hydrolysis, while being quite stable under acidic conditions.

Mechanic-chemical degradation of bioplastics occurs within film processing, thus posing some limitations to their ultimate performance, once transformed into films for packaging, and to the possibility of recycling. However, the addition of antioxidant additives shows promising potential in avoiding this type of degradation pathway.

A more recent strategy for limiting some drawbacks of bioplastics is the preparation of multilayer polymeric systems, usually achieved by film blowing of different biodegradable polymers through coextrusion. Being an emerging trend, only few papers report on this approach and several limitations still need to be solved, such as unsatisfactory interlayer adhesion, poor mechanical performance, and thermal degradation of the polymers during film-blowing operations.

## Figures and Tables

**Table 1:** Synoptic table of the discussed studies about multilayer films.

Bioplastic(s)	Composition/Layout	Additives	Processing	Main Outcomes	Ref.
Sugar-palm starch (SPS) and polylactic acid (PLA)	Bilayer		Solvent-casting	Lack of interface adhesion between the two layers	[114]
PLA-soy protein (SPI)	bilayer		Solvent-casting	Adequate layer-layer adhesion	[115]
Chitosan (CS) and whey protein (WP)	bilayer		Solution coating	Good transparency, improved mechanical performance, better water barrier properties	[116]
Poly(hydroxybutyrate-co-valerate) (PHBV)/zein/PHBV PHBV/zein/alginate	Three-layer	CNMA	Hot pressing +electrospinning	Good interlayer adhesion; reduced transparency	[17]
PLA/polyvinyl pyrrolidone (PVP)/GO/PVP/PLA	multilayer		Hot pressing, GO layer obtained by solvent method	Excellent barrier properties to H_2_O and O_2_	[16]
PHBV/PBAT	bilayer		Co-extrusion	Good mechanical properties; poor interfacial adhesion	[93]
